# CXCR1 and its downstream NF-κB inflammation signaling pathway as a key target of Guanxinning injection for myocardial ischemia/reperfusion injury

**DOI:** 10.3389/fimmu.2022.1007341

**Published:** 2022-10-17

**Authors:** Guangxu Xiao, Jiaxu Liu, Huanyi Wang, Shuang He, Jianwei Liu, Guanwei Fan, Ming Lyu, Yan Zhu

**Affiliations:** ^1^ State Key Laboratory of Component-based Chinese Medicine, Tianjin University of Traditional Chinese Medicine, Tianjin, China; ^2^ Tianjin Haihe Laboratory, Tianjin University of Traditional Chinese Medicine, Tianjin, China; ^3^ School of Integrative Medicine, Tianjin University of Traditional Chinese Medicine, Tianjin, China; ^4^ National Clinical Research Center for Chinese Medicine Acupuncture and Moxibustion, First Teaching Hospital of Tianjin University of Traditional Chinese Medicine, Tianjin, China; ^5^ Institute of Chinese Materia Medica, China Academy of Chinese Medical Sciences, Beijing, China

**Keywords:** Guanxinning injection, Myocardial ischemia/reperfusion injury, Danshensu, CXCR1, IL-8 signaling pathway

## Abstract

Guanxinning Injection (GXNI) is used clinically to treat cardiac injury, but its active components and mode of action remains unclear. Therefore, a myocardial ischemia/reperfusion injury (MIRI) model-based integrated strategy including function evaluation, RNA-seq analysis, molecular docking, and cellular thermal shift assay (CETSA) was employed to elucidate the effect and mechanism of GXNI and its main ingredient on cardiac injury. These results revealed that GXNI significantly improved cardiac dysfunction and myocardial injury in I/R mice. RNA-seq analysis clarified that CXCR1-mediated interleukin-8 pathway played a critical role in MIRI. Molecular docking screening identified danshensu (DSS) as the major active components of GXNI targeting CXCR1 protein, which was confirmed in an oxygen-glucose deprivation/reoxygenation-induced cardiomyocytes damage model showing that GXNI and DSS reduced the protein expression of CXCR1 and its downstream NF-κB, COX-2, ICAM-1 and VCAM-1. CETSA and isothermal dose-response fingerprint curves confirmed that DSS combined with CXCR1 in a dose-dependent manner. Furthermore, GXNI and DSS significantly decreased the expression levels of IL-6, IL-1β and TNF-α and the number of neutrophils in post I/R myocardial tissue. In conclusion, this study revealed that GXNI and its active components DSS exert inhibitory effects on inflammatory factor release and leukocyte infiltration to improve I/R-induced myocardial injury by down-regulating CXCR1-NF-κB-COX-2/ICAM-1/VCAM-1 pathway.

## Introduction

Although the prognosis and treatment have improved significantly in the past ten years, cardiovascular diseases, including acute myocardial infarction (AMI), are still among the leading causes of death in the world (1–3). After myocardial ischemia, timely reperfusion strategy enhances the myocardial rescue effect of most patients with AMI, but myocardial ischemia/reperfusion injury (MIRI) can also cause myocardial cell death and lead to arrhythmia, myocardial remodeling and heart failure (4–6). Therefore, it is still urgent need to develop myocardial protective strategies to effectively reduce MIRI.

Traditional Chinese medicine (TCM) has been used for thousands of years in the prevention and treatment of cardiovascular diseases (7, 8). Compared with monomer drugs with single target, the multi-component and multi-target TCM are more advantageous for the treatment of complex diseases such as AMI and stroke (9–11). Guanxinning injection (GXNI) is a Sino Food and Drug Administration (SFDA)-approved Chinese Materia Medica and is widely prescribed for coronary artery disease (CAD) indications in China. It is composed of Danshen (*Radix Salvia miltiorrhiza*) and Chuanxiong (*Rhizoma Ligusticum chuanxiong*) with the effect of “Blood-activating and stasis-removing” according to TCM theory. Modern pharmacological studies have shown that Danshen has a wide range of pharmacological effects, mainly but not limited to cardiovascular therapeutics (12, 13). For example, it inhibits IGF-II receptor signaling pathway by estrogen receptor, so as to play an anti-apoptotic role in cardiomyocytes (14); it exerts an anti-cardiac hypertrophy effect *via* inhibiting the Leu27IGF-II-IGF2R signaling pathway (15); it plays an anti-cancer effect by down-regulating the mitogen-activated protein kinase (MAPK)/activator protein-1 (AP-1) signaling pathway (16); it improve cerebral vascular endothelial damage caused by cerebral ischemia/reperfusion injury by inhibiting the Src signaling pathway (17), and so on. Chuanxiong, another Traditional Chinese Medicine commonly used to treat cardio-cerebrovascular diseases, its main active ingredients are phthalides and alkaloids (18). Similarly, Chuanxiong has extensive pharmacological effects, such as anti-cerebral ischemia (19), anti-myocardial ischemia (20), anti-thrombosis (21), anti-inflammatory (22), anti-oxidation (23), and anti-asthma effects (24). Moreover, the research of Chuanxiong reveals that Chuanxiong increases the expression of VEGF after myocardial infarction to promote angiogenesis (25), regulates autophagy *via* activating the PI3K/Akt/mTOR signaling pathway (20), and can also improve angina by inhibiting acid-sensitive ion channels (26). Although the above research results provide the possibility for GXNI in the treatment of AMI, its therapeutic effect is still unknown.

In this study, we evaluated the therapeutic effects of GXNI through the mouse model with myocardial ischemia/reperfusion injury (MIRI), and revealed the potential mechanisms of GXNI in the treatment of AMI in combination with RNA-seq and network pharmacological analysis, which revealed that CXCR1 plays a key role in it. The discovery has greatly attracted our research interest and attention. It is well known that there are differences between humans and rodents. The hIL-8/CXCL8 exerts biological functions in human by activating human CXCR1 (hCXCR1) and human CXCR1 (hCXCR2), but the functional identification of murine CXCR1 (mCXCR1) in mice was not smooth at first. Only one functional ELR-CXC receptor has been previously identified in mice and characterized as a homologue of hCXCR2, namely, mCXCR2 (27, 28). Because mCXCR2 has high affinity for hCXCL8, mCXCL1 and MIP-2, and causes neutrophil recruitment and activation, it is considered to be the receptor responsible for the full function of hCXCR1 and hCXCR2 in humans (29, 30). Later, although it was found that mCXCR1 was expressed in basement membrane cells, peripheral monocytes, CD4^+^ and CD8^+^ T cells and some lymphocytes of mice, it is regrettable that its biological function has not been confirmed (31, 32). This also led people to think that mCXCR1 was a non-functional receptor. Excitingly, as research progressed, it has been found that mCXCR1 is specifically activated by mGCP-2, hGCP-2/CXCL6 and hIL-8/CXCL8, and exerts critical role in collagen-induced arthritis in mice (33, 34). This finding demonstrates that mCXCR1 is a functional receptor and opens a “door” for biological research on CXCR1/2 using mouse models in the future. In recent years, a variety of biological functions of CXCR1 *in vivo* have been discovered, and it is most reported in cancer (35–38). Endogenous CXCR1 can not only directly interfere with the resistance of cancer cells to chemotherapeutic drugs, but also be an active regulator of cancer cell metastasis (39). With the deepening of research, CXCR1 is regarded as a novel therapeutic target in various cancer treatments (37, 38, 40). Furthermore, CXCR1 is also involved in the progression of coronary artery disease caused by decreased myocardial blood supply, and plays an increasingly synaptic role (41). The increased expression of CXCR1 can predict the occurrence of immune activation and remodeling in patients with heart failure, and may even be an effective marker for the diagnosis of AMI (42, 43). In this study, we not only clarified that CXCR1 is a key target for the treatment of AMI, but also found for the first time that GXNI exerts a therapeutic effect on AMI *via* inhibiting the activation of CXCR1 and its downstream pathways in myocardial tissue. The direct interaction between GXNI and CXCR1 was further evaluated by molecular docking and CETSA, and Danshensu, the active ingredient of GXNI in regulating CXCR1, was identified.

## Materials and methods

### Drugs and reagents

Guanxinning injection (SFDA drug approval number Z13020779) was provided by China Shineway Pharmaceutical Group Ltd. (Shijiazhuang, China, batch number 171122B2) (44, 45). 2, 2, 2-Tribromoalcohol was purchased from Sigma (T48402, St. Louis, MO, United States). Metoprolol-tartrate (YZ-100084), 2, 3, 5-Triphenyl-2H-Tetrazolium Chloride (TTC, G3005), Bicinchoninic acid protein concentration assay kit (BCA, PC0020), High-efficiency RIPA tissue/cell lysate (R0010), Tris Buffered saline Tween (TBS-T, T1081), Tris-Glycine Running Buffer (T1070) and Western blot Transfer Buffer (D1060) were purchased from Solarbio (Beijing, China). Pierce™ ECL Western Blotting Substrate (32109) was purchased from Thermo Fisher Scientific (Waltham, United States). SDS-PAGE Protein Loading Buffer (P0015L) and Hematoxylin and Eeosin (H&E) staining kit (C01055) were purchased from Beyotime Biotechnology (Shanghai, China). Polyvinylidene Difluoride (PVDF) Blotting Membrane was purchased from GE Healthcare Life Science (Pittsburgh, United States). Anti-CXCR1 antibody (bs-1009R) and anti-VCAM-1 antibody (bs-0396R) were purchased from Bioss (Beijing, China). Anti-COX2 antibody (12282S) was purchased from Cell Signaling Technology (Danvers, United States). Anti-NFkB (AF5006) and anti-ICAM-1 (AF6088) antibodies were purchased from Affinity Biosciences (Liyang, China). Anti-GAPDH (YM3215) and HRP Goat anti-Rabbit (RS0002) antibodies were purchased from Immunoway (Plano, United States). Transcriptor First Strand cDNA Synthesis Kit was purchased from Roche (04897030001, Mannheim, Germany). Bestar™ qPCR MasterMix was purchased from DBI Bioscience (DBI-2043, Shanghai, China). Mouse ELISA kit for cTNT (ZC-38814), IL-6 (ZC-37988), IL-1β (ZC-37974) and TNF-α (ZC-39024) were purchased from ZCIBIO Technology Co., Ltd (Shanghai, China). Creatine Kinase Assay Kit (XM0140), Creatine Kinase-MB Isoenzyme Assay Kit (XM0240), Lactate Dehydrogenase Assay Kit (XM0340) and Aspartate Aminotransferase Assay Kit (GM0240) were purchased from WANTAI DRD Co., Ltd (Beijing, China). Reparixin (HY-15251) was purchased from MedChemExpress (New Jersey, United States).

### Experimental animals

All animal experiment protocols were designed in accordance with the principles of the Basel Declaration and recommendations of the Care and Use of Laboratory Animals promulgated by the Ministry of Science and Technology of China and were implemented after approval by the Laboratory Animal Ethics Committee of Tianjin University of Traditional Chinese Medicine (License number: TCM-LAEC2020107). Wild-type male C57BL/6J mice aged 8-10 weeks were purchased from Beijing Vital River Laboratory Animal Technology Co., Ltd. (Beijing, China, Certificate number: SCXK Jing 2017-0005). Mice were reared in cages where they could eat and drink freely under the condition of 22°C ± 2°C and relative humidity of 40% ± 5%, and maintained a 12-hrs light/dark cycle.

### Mouse model of MIRI and drug administration

The MIRI model was established as previously described (46). Briefly, the mice were anesthetized and intubated to link a ventilator. Thoracotomy was performed to expose the heart and the left anterior descending (LAD) coronary artery near the left atrial appendage (LAA) was ligated for 30 min with 7-0 monofilament suture. After 30 min of myocardial ischemia, the suture was untied to restore blood flow and reperfusion for 24 hrs. ST-segment elevation on an electrocardiogram monitor represented the success of MIRI model operation. The mice were randomly divided into six groups: sham, I/R (saline), GXNI 1 mL/kg, GXNI 3 mL/kg, GXNI 9 mL/kg, and positive control drug Metoprolol (10 mg/kg), in which the sham mice were subjected to thoracotomy, and the suture thread was passed through the ligation position but not ligated. All groups were injected with drugs or normal saline through tail vein within 10 min of myocardial ischemia.

### Echocardiographic measurement

Twenty-four hrs after reperfusion, the left ventricular function and coronary blood flow of the mouse heart were measured using an ultra-high resolution small animal ultrasound imaging system (Vevo 2100, VisualSonics, Toronto, ON, Canada). The fully anesthetized mice were fixed on a heated imaging platform, and the appropriate amount of chelating agent was coated on the limbs and chest of the mice for echocardiography and electrocardiogram detection. The left ventricular function was measured in M-mode, and the indexes were as follows: left ventricular (LV) ejection fraction (EF), LV fractional shortening (FS), LV septal thickness at diastole (LVSd), LV septal thickness at systole (LVSs), LV internal diameter at diastole (LVIDd), LV internal diameter at systole (LVIDs), LV posterior wall at diastole (LVPWd), LV posterior wall at systole (LVPWs), LV systole volume (LV Vols), LV diastole volume (LV Vold), heart rate and LV mass. Coronary blood flow was determined using Color Doppler mode, which was evaluated by aortic valve (AV) peak velocity, AV peak pressure and aorta velocity-time integral mean velocity (AoV VTI).

### Quantification of myocardial infarction size

At 24 hrs after myocardial ischemia-reperfusion, the mice were euthanized and their hearts were removed immediately, washed with normal saline, placed in a heart matrix device and cut into 4 continuous slices (2 mm thick), and then incubated in 2% TTC solution for 10 min in the dark at 37°C. The staining results were photographed and the area of myocardial infarction was measured by ImageJ software (National Institutes of Health, Bethesda, MD, United States). Myocardial infarction rate (%) is calculated as infarct size (white area)/total area of myocardial cross section × 100%.

### Measurement of biochemical parameters

After the mice were anesthetized by isoflurane, a U-shaped incision was made in the abdomen using ophthalmic scissors. After the abdominal aorta was exposed, blood was drawn utilizing a 1-mL syringe and centrifuged at 3000 rpm for 15 min to separate serum. The mice were then euthanized by cervical dislocation under anesthesia. The concentrations of LDH, AST, CK-MB and CK in mouse serum were measured by automatic biochemical analyzer (TBA-40F, Toshiba, Tokyo, Japan) and ELISA kit was used to determine the concentration of cTNT in the serum according to the manufacturer’s instructions.

### RNA sequencing

A library of total RNA extracted from the heart tissue was first constructed. To ensure the quality and reliability of data processing, reads containing adapter and ploy-N, and low-quality reads in the original Illumina sequencing data were deleted. Then the HISAT2 alignment software (The Johns Hopkins University, Baltimore, Maryland, United States) was utilized to compare the processed reads with the Mus musculus genome, and the featureCounts tool of subread software (The Walter and Eliza Hall Institute of Medical Research, Parkville, Australia) was used to quantify gene expression levels. Finally, differential expression analysis between groups was performed using DESeq2 software.

### Ingenuity^®^ pathway analysis

Core analysis of Ingenuity Pathway Analysis (IPA) software (QIAGEN, Denmark) was used to analyze the significant differential expressed genes (DEGs, fold change ≥ 1.5 and P-value ≤ 0.01) in RNA-seq. The significance of the analysis results was evaluated by the ratio of the number of molecules mapped to the data set in the pathway divided by the total number of genes in the pathway and the P-value calculated by Fisher’s exact test.

### Real-time reverse transcription polymerase chain reaction assay

Total RNA was extracted from heart tissue using TRIzol™ Reagent. The cDNA was obtained by reverse transcription using Transcriptor First Strand cDNA Synthesis Kit, added to the PCR strip tubes with Bestar™ qPCR MasterMix and primers, and amplified in a real-time PCR system (LightCycler^®^480, Roche, Germany) to detect its mRNA expression level. The genes to be detected in this experiment include C-X-C motif chemokine receptor 1 (*Cxcr1*), C-X-C motif chemokine receptor 2 (*Cxcr2*), TNF receptor associated factor 6 (*Traf6*), mitogen-activated protein kinase 10 (*Mapk10*), ras homolog family member V (*Rhov*), cytochrome c oxidase subunit II (*Cox2*), using glyceraldehyde 3-phosphate dehydrogenase (*Gapdh*) as a standard. Primers for the above genes (sequences listed in **Table 1**) were synthesized by Sangon Company (Shanghai, China).

**Table 1:** Primer sequences.

Primer name Primer sequence (5’–3’)	
* Cxcr1* sense TCTGGACTAATCCTGAGGGTG * Cxcr1* antisense GCCTGTTGGTTATTGGAACTCTC * Cxcr2* sense ATGCCCTCTATTCTGCCAGAT * Cxcr2* antisense GTGCTCCGGTTGTATAAGATGAC * Traf6* sense AAAGCGAGAGATTCTTTCCCTG * Traf6* antisense ACTGGGGACAATTCACTAGAGC * Mapk10* sense CCATGTCTGTGTTCTTTCTCACG * Mapk10* antisense TTGGTTCCAACTGTGAAGAGTC * Rhov* sense CGCTATCGGCCTACAGCAC * Rhov* antisense CGGGTAGCAGAGAGAACGAA * Cox2* sense TGAGCAACTATTCCAAACCAGC * Cox2* antisense GCACGTAGTCTTCGATCACTATC

### Western blotting

The heart tissue was lysed using a high-efficiency RIPA tissue/cell lysate, and protein concentration in the supernatant was determined by BCA kit. The protein samples were separated with SDS-polyacrylamide gel electrophoresis and transferred to PVDF membrane. The membrane was blocked, incubated with the primary antibody overnight at 4°C, washed with TBS-T and incubated with the corresponding secondary antibody at room temperature for 2 hrs. Finally, the target protein was captured in the gel imaging system after the ECL Western Blotting substrate development. The protein expression levels of CXCR1, NF-κB, COX-2, ICAM-1 and VCAM-1 were analyzed by ImageJ and standardized to GAPDH.

### Hematoxylin and eosin staining

H&E staining was performed as previously described (46). Briefly, heart tissue was fixed in 4% paraformaldehyde, dehydrated by automatic dehydrator (Excelsior, Thermo Fisher Scientific, Ltd., Waltham, United States) and embedded in paraffin. Next, the embedded tissue was cut into 5-μm thick slices using a manual slicer (HM355S, Thermo Fisher Scientific, Ltd.). Finally, the sections were placed in an automatic slice stainer (Gemini, Thermo Fisher Scientific, Ltd., United States) for H&E staining. After staining, the slices were mounted and photographed *via* an automated quantitative pathology imaging system (Vectra 3, PerkinElmer Inc., Boston, United States) to display the pathological and structural changes and the infiltration of neutrophils in heart tissue.

### Immunohistochemistry

After paraffin removal, heart tissue sections were rinsed 3 times in PBS-T solution and incubated with 3% peroxide-methanol for 10 min to block the endogenous peroxidase. The sections were then blocked with 10% bovine serum albumin solution at 37°C for 1 hr, incubated with primary antibody (anti-CXCR1, 1:100 diluted in blocking solution) for 2 hrs, washed 3 times with PBS-T solution for 5 min, and incubated with the corresponding secondary antibody at 37° C for 1 hr. The immunoreactivity of CXCR1 protein was visualized by 3,3’-diaminobenzidine tetrahydrochloride hydrate, and stained with hematoxylin and fixed. Finally, the expression of CXCR1 was quantified by an automated quantitative pathology imaging system (Vectra 3, PerkinElmer Inc.).

### Immunohistofluorescence

Immunohistofluorescence staining was performed as previously described (47). Briefly, the deparaffinized sections were placed in citrate buffer for antigen retrieval. After washing, the slices were incubated in 3% hydrogen peroxide solution at room temperature for 20 min. After antigen blocking was completed, the diluted primary antibody (myeloperoxidase polyclonal antibody, 1:100) was added to the sections and incubated overnight at 4°C. The next day, the fluorescent secondary antibody Alexa Fluor 488 (1:200, A11012, Thermo Fisher Scientific) and DAPI nucleic acid staining solution (1:150, D1306, Thermo Fisher Scientific) were incubated in dark after tissue sections were washed with PBS-T. After sealing the slide with sealant, neutrophils in myocardial tissue were imaged and quantified by Vectra 3 pathology imaging system.

### Molecular docking

Chemical structures of the GXNI ingredients were downloaded from ChemSpider website (http://www.chemspider.com/), while the X-ray 3D crystal structure of CXCR1 protein was obtained from RCSB PDB databank (https://www.rcsb.org/). Both were introduced into Discovery Studio 2019 Client for analysis and display of the interaction relationship. In the “Receptor-Ligand Interactions” module of the software, a CHARMm-based molecular dynamics scheme (CDOCKER) was used to run the interaction between the GXNI ingredients and CXCR1, which were shown as 2D and 3D interaction diagrams.

### Cell culture

Mouse HL-1 cardiomyocytes or human AC-16 cardiomyocytes were cultured in 10-cm petri dish in Dulbecco’s modified eagle medium (DMEM) containing 1% penicillin-streptomycin (PS) and 10% fetal bovine serum (FBS) and incubated at 37°C and 95% air/5% CO_2_.

### Establishment of OGD/R model and drug administration

Cellular OGD/R injury model was established as previously described (47). In short, Mouse cardiomyocyte cell line HL-1 cells were cultured in glucose-free medium and placed in a hypoxic chamber containing 95% N_2_ and 5% CO_2_ for 150 min. Then, it was replaced with normal complete medium and cultured in normal oxygen atmosphere containing 5% CO_2_ at 37°C for 24 hrs. In order to study whether DSS can improve the OGD/R damage, the HL-1 cells were divided into 6 groups: Control (normal growth condition), OGD/R (HL-1 cells were treated with OGD/R), DSS doses (HL-1 cells were cultured in complete medium containing 0.3 μM, 1 μM and 3 μM DSS for 24 hrs after OGD), and GXNI (HL-1 cells were cultured in complete medium containing 1 μL/mL GXNI for 24 hrs after OGD). In addition, HL-1 cells were divided into 6 groups to study whether DSS can protect cardiomyocytes by inhibiting CXCR1-NF-κB-COX-2 pathway, including Control group, OGD/R, DSS (3 μM), GXNI (1 μL/mL), Reparixin (100 nM) and DSS + Reparixin (3 μM +100 nM).

### Immunocytofluorescence

HL-1 cells on the 96-well plate were fixed with 4% paraformaldehyde solution and blocked with 5% FBS for 2 hrs, incubated with primary antibody (CXCR1, 1:200; NF-κB, 1:200; VCAM-1, 1:200; ICAM-1, 1:200 and COX-2, 1:800) overnight at 4°C, washed with PBS-T, and incubated with the corresponding fluorescent secondary antibody and Hoechst 33342 for 2 hrs at room temperature. Finally, after washing the cells with PBS-T in the dark, Operetta high-content screening system (PerkinElmer Inc., USA) was used to detect the cell number and fluorescence intensity of HL-1.

### Cell viability assay

HL-1 was inoculated in a 96-well plate at a density of 10^4^ cells/well and cultured in complete medium containing different concentrations of DSS (0.3 µM, 1 µM, 3 µM, 10 µM, 30 µM, 100 µM, and 300 µM) for 24 hrs. Ten µL of CCK-8 solution was then added to each well, incubated at 37°C for 4 hrs, and cell viability was determined by the absorbance at 450 nm with a microplate reader.

### Cellular thermal shift assay

Cellular thermal shift assay was performed as described previously (48, 49). Briefly, mouse HL-1 cardiomyocytes cultured in a 10-cm petri dish were collected and lysed by repeatedly frozen and thawed in liquid nitrogen, and the protein in supernatant was collected by centrifugation. The hCXCR1 protein was obtained from human AC-16 cardiomyocytes by the same procedure. The supernatant was divided, and treated with physiological saline or drugs (5 μL/mL GXNI or 20 μM Danshensu) for 30 min at room temperature. After the reaction, each sample was aliquoted into ten portions and heated in a thermo cycler for 3 min at the following temperature: 37°C, 41°C, 45°C, 49°C, 53°C, 57°C, 61°C, 65°C, 69°C, and 73°C. Next, the samples were centrifuged at 20,000 g for 20 min, and the same amount of supernatant was collected into a 1.5 ml EP tube. After adding an appropriate amount of loading buffer, the samples were heated at 95°C for 10 min and WB experiments were performed. Finally, the CETSA curves were obtained by ImageJ analysis.

### Isothermal dose-response

Isothermal dose-response assay was performed as described previously (48). Briefly, different concentrations of Danshensu (0, 1, 2, 5, 10, and 20μM) were added into divided HL-1 cell lysates and incubated at room temperature for 30 min. Then, the samples were heated at 61°C for 3 min in a thermo cycler and centrifuged at 20000 g for 20 min to remove the precipitation. After adding the loading buffer, the samples were heated at 95°C for 10 min and WB experiments were performed. Finally, the ITDRF_CETSA_ curve was acquired by ImageJ software.

### Receptor activation assay

HL-1 or AC-16 cells were inoculated in 96-well plates at a density of 10^4^ cells/well. To investigate whether DSS inhibits the activation of CXCR1 and CXCR2 by recombinant protein IL-8, cells were divided into 5 groups: Control, IL-8 (HL-1 or AC-16 cells cultured in complete medium containing 100 nM IL-8), IL-8+DSS (HL-1 or AC-16 cells cultured in complete medium containing 100 nM IL-8 and 1 μM DSS), IL-8+GXNI (HL-1 or AC-16 cells cultured in complete medium containing 100 nM IL-8 and 1 μL/mL GXNI), IL-8+Reparixin (HL-1 or AC-16 cells cultured in complete medium containing 100 nM IL-8 and 100 nM Reparixin). After 24 hrs of culture, cells were fixed with 4% paraformaldehyde solution and blocked with 5% FBS for 2 hrs, incubated with primary antibody (CXCR1, 1:200; CXCR2, 1:200) overnight at 4°C, washed with PBS-T, and incubated with the corresponding fluorescent secondary antibody and Hoechst 33342 for 2 hrs at room temperature. Finally, after washing the cells with PBS-T in the dark, the fluorescence intensity of CXCR1 and CXCR2 was evaluated using the Operetta high-content analysis system.

### Statistical analysis

Data were expressed as mean ± SEM. GraphPad Prism 7 software (GraphPad Software, Inc., La Jolla, CA, United States) was used for statistical analysis. The student’s two-tailed t-test was utilized for comparison between two groups, while multiple groups comparison was performed with one-way analysis of variance (ANOVA). A value of P < 0.05 was defined as statistically significant.

## Results

### GXNI ameliorated the cardiac function in MIRI mice

**Figure 1:**
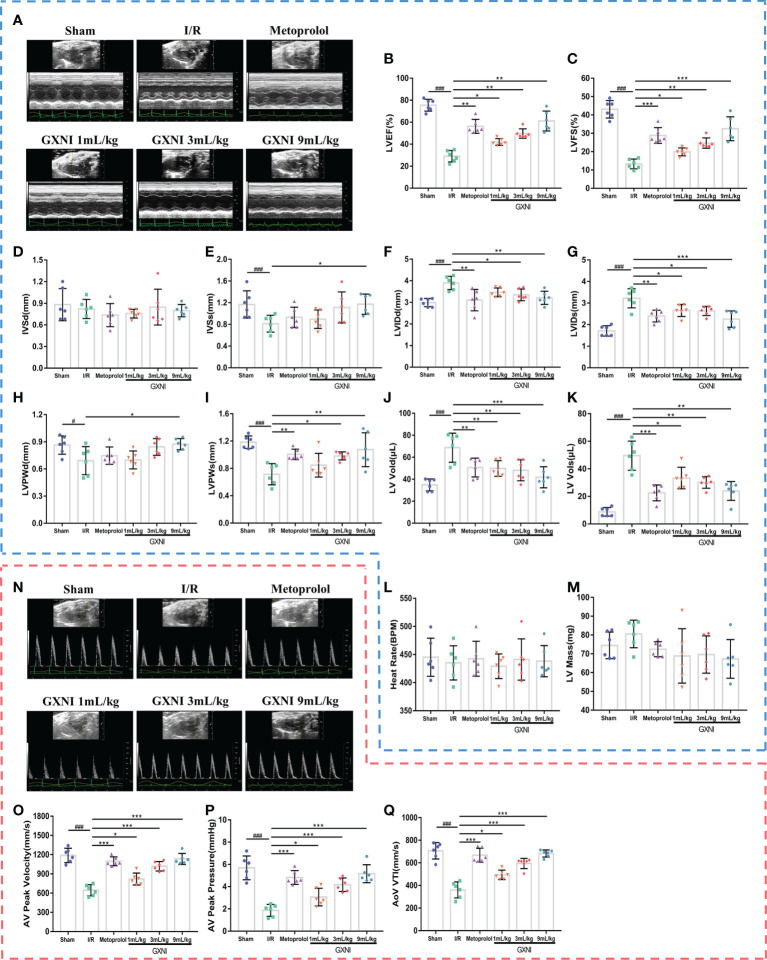
The effect of GXNI on echocardiographic characteristics of cardiac function and coronary blood flow in I/R mice. After 30 min of ischemia and 24 hrs of reperfusion, the cardiac function and coronary blood flow of mice in each group were evaluated. **(A)** Representative echocardiographic images of sham group, I/R group, metoprolol group and different GXNI dose (1 mL/kg, 3 mL/kg, 9 mL/kg) groups. Quantification of echocardiographic changes in cardiac function in different groups detected in M mode: **(B)** LVEF %, **(C)** LVFS %, **(D)** LVSd, **(E)** LVSs, **(F)** LVIDd, **(G)** LVIDs, **(H)** LVPWd, **(I)** LVPWs, **(J)** LV Vold, **(K)** LV Vols, **(L)** heart rate and **(M)** LV mass. **(N)** Representative echocardiography images of coronary blood flow images of each group. **(O)** AV peak velocity, **(P)** AV peak pressure and **(Q)** AoV VTI were determined in color Doppler mode. Values were expressed as mean ± SEM (n = 6). ^###^ *P* < 0.001, ^#^ *P* < 0.05 vs. sham group, ^*^ *P* < 0.05, ^**^ *P* < 0.01, ^***^ *P* < 0.001 vs. I/R group (LV, left ventricular; EF, ejection fraction; FS, fractional shortening; LVSd, LV end diastolic septal thickness; LVSs, LV septal thickness at end systole; LVIDd, LV internal dimensions at diastole; LVIDs, LV internal diameter systole; LVPWd, LV posterior wall diastole; LVPWs, LV posterior wall systole; LV Vold, LV diastole volume; LV Vols, LV systole volume; AV, aortic valve; AoV VTI, aorta velocity–time integral mean velocity).

After 24 hrs of reperfusion, cardiac function was evaluated by echocardiography **Figure 1A**. Compared with the sham operation, MIRI significantly decreased LVEF% (**Figure 1B**), LVEF% (**Figure 1C**), LVSs (**Figure 1E**), LVPWd (**Figure 1H**) and LVPWs (**Figure 1I**), while increased LVIDd (**Figure 1F**), LVIDs (**Figure 1G**), LV Vold (**Figure 1J**) and LV Vols (**Figure 1K**), without significantly changing LVSd (**Figure 1D**), heart rate (**Figure 1L**) and LV mass (**Figure 1M**). This indicated that severe cardiac dysfunction occurred in mice after 30 min of myocardial ischemia and 24 hrs of reperfusion. On the other hand, different doses of GXNI (1 mL/kg, 3 mL/kg and 9 mL/kg) and the positive control drug metoprolol all markedly improved LVEF% (**Figure 1B**), LVFS% (**Figure 1C**), LVIDs (**Figure 1G**), LV Vold (**Figure 1J**) and LV Vols (**Figure 1K**) in MIRI mice. GXNI at doses of 9 mL/kg also ameliorated LVSs (**Figure 1E**), LVIDd (**Figure 1F**), LVPWd (**Figure 1H**) and LVPWs (**Figure 1I**) in MIRI mice. The cardiac function parameters LVIDd (**Figure 1F**) and LVPWs (**Figure 1I**) in the 3 mL/kg GXNI- and metoprolol-treated mice were also significantly improved compared with the MIRI group. Furthermore, compared with the MIRI group, neither GXNI nor metoprolol significantly affected LVSd (**Figure 1D**), heart rate (**Figure 1L**), and LV Mass (**Figure 1M**). Color Doppler of coronary blood flow results showed that the AV peak velocity, AV peak pressure and AoV VTI of the MIRI mice were remarkably lower than those in the sham group (**Figures 1N–Q**). However, the decrease of AV peak velocity, AV peak pressure and AOV VTI induced by MIRI were effectively improved by GXNI (1 mL/kg, 3 mL/kg and 9 mL/kg) and metoprolol (**Figures 1N–Q**). In conclusion, these results suggested that GXNI significantly alleviated cardiac dysfunction caused by myocardial ischemia/reperfusion injury.

### GXNI attenuated myocardial injury in I/R mice

**Figure 2:**
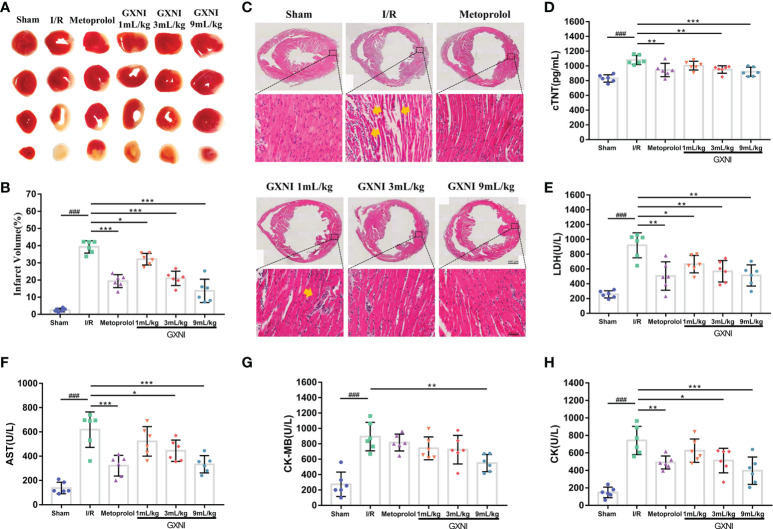
GXNI reduced the volume of myocardial infarction in I/R mice. After 30 min of myocardial ischemia and 24 hrs of reperfusion, the myocardial tissue of mice in each group, including sham, I/R, metoprolol, GXNI 1 mL/kg, GXNI 3 mL/kg and GXNI 9 mL/kg, were stained with TTC. **(A)** Representative images of TTC staining for each group. Normal myocardial tissue was in red, while the infarcted area was in white. **(B)** Quantification of infarct volume in each group. **(C)** Representative pictures of H&E staining results of myocardial tissue, in which myocardial injury was indicated by yellow arrow. The upper part was the panoramic scanning image of the cross-section of the cardiac tissue in each group (Scale bar = 600 μm), and the lower part was the corresponding local image (Scale bar = 40 μm). **(D–H)** The levels of LDH, AST, CK, CK-MB and cTNT in serum of mice in different groups were measured by automatic biochemical analyzer and ELISA kit. Values were expressed as mean ± SEM (n = 6). ^###^ *P* < 0.001 vs. sham group, ^*^ *P* < 0.05, ^**^ *P* < 0.01, ^***^ *P* < 0.001 vs. I/R group.

To evaluate the protective effect of GXNI on MIRI mice, TTC staining, H&E staining and myocardial zymogram detection were performed. After 30 min of ischemia and reperfusion for 24 hrs, the infarct volume of mice in the MIRI group was significantly increased compared with that of the sham group, while the intervention of GXNI and metoprolol obviously reduced the infarct volume (**Figures 2A, B** **)**. H&E staining results also revealed that MIRI injury resulted in an obvious cell structural disarray and necrosis and leukocyte infiltration in myocardial tissue, which were indicated by yellow arrows in **Figure 2C**. In contrast, GXNI treatment also improved the histopathological characteristics of damaged tissues (**Figure 2C**). GXNI and metoprolol markedly reduced the elevation in LDH (**Figure 2E**), AST (**Figure 2F**), CK (**Figure 2H**) and cTNT (**Figure 2D**) levels caused by MIRI injury in mouse serum. Furthermore, 9 mL/kg GXNI also ameliorated the level of CK-MB in the serum of I/R mice (**Figure 2G**). In addition, the GXNI effects in all the parameters exhibited a dose-dependency with 9 mL/kg being more effective than 1 mL/kg or 3 mL/kg (**Figure 2**). Taken together, these results indicated that GXNI significantly alleviated MIRI-mediated myocardial damage.

### Transcriptome sequencing of heart tissue of MIRI mice treated with GXNI identified potential candidate genes

**Figure 3:**
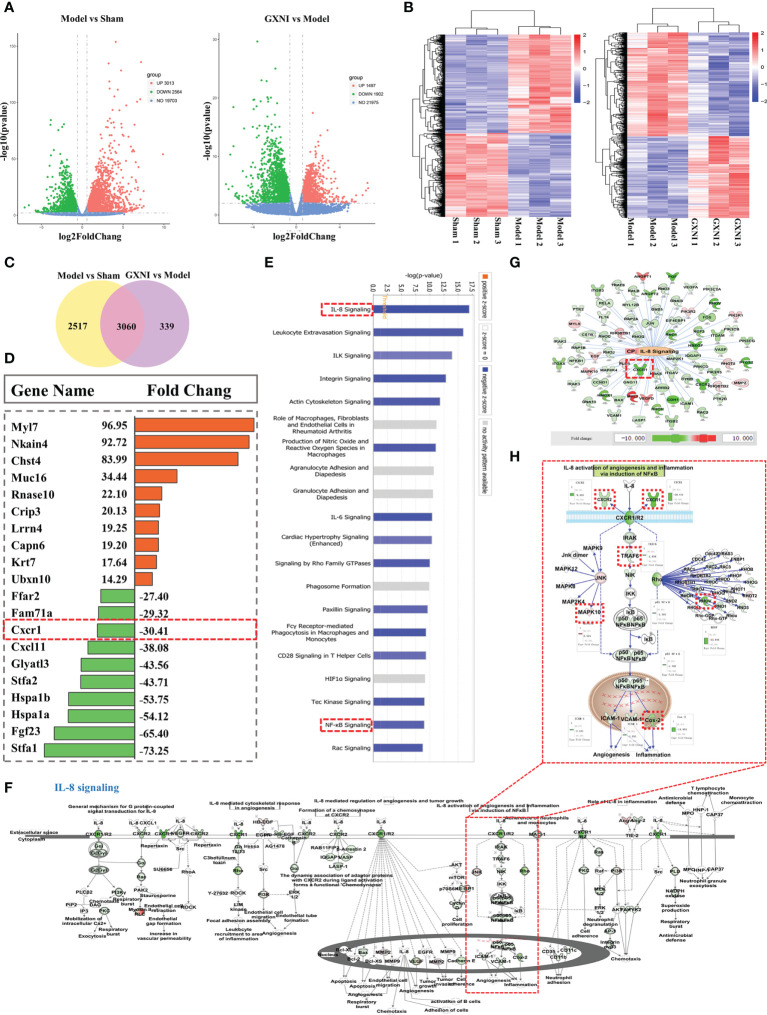
Transcriptome sequencing results and IPA analysis. **(A)** The differential gene distribution (fold change ≥ 1.5 and p value ≤ 0.01) among the sham group, I/R (Model) group and GXNI 9 mL/kg group was displayed through a volcano graph. The abscissa and ordinate represented the fold change and the significance level of the difference in expression of the gene, respectively. Red dots were the up-regulated genes, and green dots were the down-regulated genes. **(B)** Hierarchical cluster analysis among samples of sham group, I/R group and GXNI (9 mL/kg) group. Each group included three independent animals (n = 3). **(C)** Venn diagram of MIRI group vs. Sham group and GXNI group vs. MIRI group. **(D)** List of the top 10 genes with up-regulated fold change and the top 10 genes with down-regulated fold change among the 3060 DEGs shared by I/R model vs. sham and GXNI vs. I/R model. **(E)** Core analysis revealed the top 20 pathways of 3060 DEGs which were arranged in descending order according to -log (p-value), with IL-8 signal pathway being number one. **(F)** The schematic diagram of the interaction of genes in the IL-8 signaling pathway. **(G)** Gene targets that are regulated by GXNI in IL-8 signaling pathway. **(H)** A schematic diagram of gene interactions in CXCR1-NF-κB-COX-2/ICAM-1/VCAM-1 pathway. In **A–H**, GXNI-regulated genes in the transcriptome data are color labeled, with green representing down-regulation and brown representing up-regulation.

RNA-seq analysis was performed on the heart tissues of mice in the sham, MIRI and GXNI (9 mL/kg)-treated mice to determine the differentially expressed genes (DEGs) after I/R injury as well as the genes regulated by GXNI. The results showed that at a fold change ≥ 1.5 and P-value ≤ 0.01, I/R injury resulted in up-regulation of 3013 DEGs and down-regulation of 2564 DEGs in heart, while GXNI regulated 3399 DEGs in heart tissue of I/R mice, including 1497 up-regulated genes and 1902 down-regulated genes (**Figure 3A**, details listed in **Supplementary Tables S1**, **S2**). Among the 5577 DEGs altered by I/R injury, 3060 genes were regulated by GXNI, which indicates that GXNI may exert myocardial protection in MIRI mice by regulating these genes (**Figure 3C**, details listed in **Supplementary Table S3**). For this reason, our subsequent pathway analysis focused on these 3060 differentially expressed genes, and the top 10 up-regulated and the top 10 down-regulated DEGs were displayed in **Figure 3D**. In addition, to study the gene expression of each sample, the overall gene expression profiles of the hierarchical cluster of significantly differential genes between each group were shown in **Figure 3B**.

### Pathway analysis of differentially expressed genes (DEGs) regulated by GXNI in MIRI mouse heart

Since the 3,060 overlapping DEGs in I/R model vs. sham and GXNI vs. I/R model might be targets of GXNI to improve I/R damage, they were subjected to Core analysis in IPA. Top 20 canonical pathways affected by GXNI treatment after MIRI were arranged in descending order according to the -log(p-value) score in **Figure 3E**, of which the interleukin-8 (IL-8) signaling pathway ranked the first (**Figure 3E**). It is worth noting that CXCR1 not only plays a key role in the IL-8 signaling pathway (**Figures 3F, G** **)**, but also ranks among the top 10 in the down-regulated DEGs (**Figure 3D**), which indicates that CXCR1 may be an important target for GXNI to exert myocardial protection. In addition, the NF-κB signaling pathway, downstream of CXCR1 in the IL-8 signaling pathway (**Figure 3H**), was also ranked in the top 20 canonical pathways affected by GXNI treatment after MIRI (**Figure 3E**). As shown in **Figure 3H**, downstream molecules of NF-κB pathway such as COX-2, ICAM-1 and VCAM-1 were also down regulated by GXNI by 14.9, 2.5 and 1.8 folds, respectively.

### Effect of GXNI on CXCR1-NF-κB-COX-2/ICAM-1/VCAM-1 pathway in MIRI mouse heart

**Figure 4:**
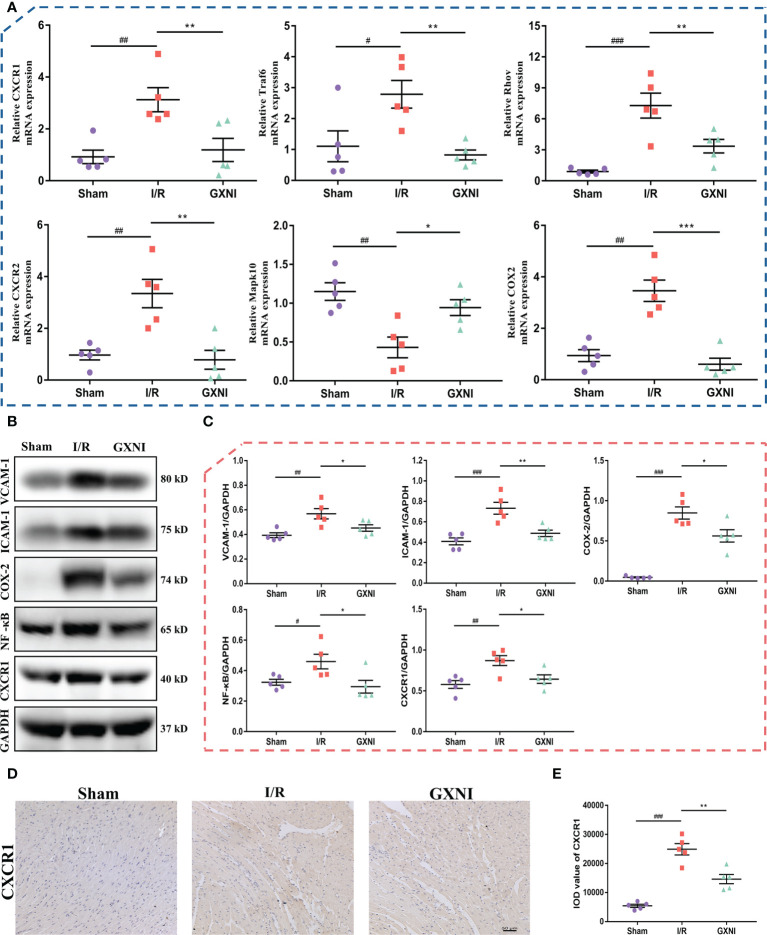
Verification of CXCR1-NF-κB-COX-2/ICAM-1/VCAM-1 pathway regulated by GXNI. **(A)** RT-PCR verification results of GXNI regulated genes in CXCR1-NF-κB-COX-2/ICAM-1/VCAM-1 pathway, including *Cxcr1*, *Cxcr2*, *Traf6*, *Mapk10*, *Rhov* and *Cox2*. **(B)** Representative blots of CXCR1, NF-κB, COX-2, ICAM-1 and VCAM-1 in the sham, I/R, and GXNI (9 mL/kg) groups. **(C)** Quantification of expression levels of CXCR1, NF-κB, COX-2, ICAM-1 and VCAM-1 proteins. **(D)** Representative pictures of immunohistochemistry staining. **(E)** Quantification of CXCR1 expression levels in cardiac tissue sections. Values were expressed as mean ± SEM (n = 5). ^#^ *P* < 0.05, ^##^ *P* < 0.01, ^###^ *P* < 0.001 vs. sham group, *P < 0.05, **P < 0.01, ***P < 0.001 vs. I/R group.

Six genes regulated by GXNI in CXCR1-NF-κB-COX-2**/**ICAM-1/VCAM-1 pathway, including *Cxcr1*, *Cxcr2*, *Traf6*, *Mapk10*, *Rhov* and *Cox2*, were verified using RT-PCR, and the results were consistent with those of transcriptome data (**Figure 4A**). In addition, the Western Blot results indicated that 9 mL/kg of GXNI reduced the expression of the key proteins in the pathway, such as CXCR1, NF-κB, COX-2, ICAM-1 and VCAM-1 in the heart tissues of MIRI mice (**Figures 4B, C** **)**. Further immunohistochemistry staining determined that consistent with the results of RT-PCR and WB, GXNI reversed the increase in the expression level of CXCR1 caused by MIRI. In addition, it also revealed that the expression location of CXCR1 was mainly on cardiomyocytes (**Figures 4D, E** **)**. In conclusion, the combination of transcriptome analysis and subsequent RT-PCR, WB and IHC verification revealed that GXNI has a significant therapeutic effect on acute myocardial infarction in mice mainly *via* inhibiting the CXCR1-NF-κB-COX-2/ICAM-1/VCAM-1 pathway.

### Identification of CXCR1-interacting GXNI ingredients by molecular docking screen

**Figure 5:**
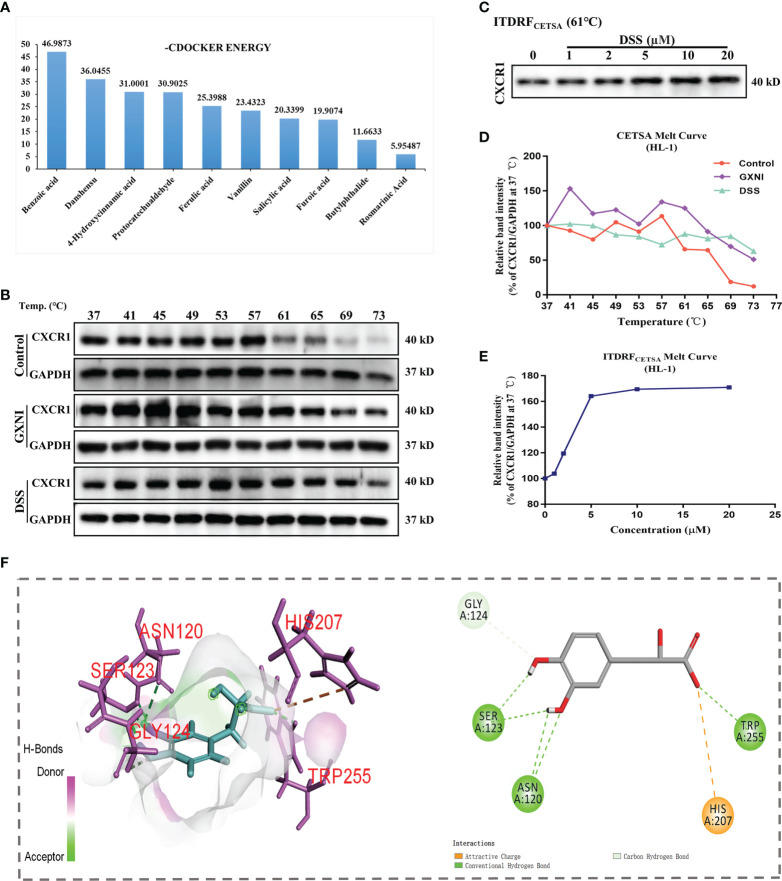
Ingredients in GXNI that bind to CXCR1. Structural-based binding prediction of GXNI ingredients to CXCR1 by molecular docking. **(A)** The score of -CDOCKER ENERGY of ingredients in GXNI combined with CXCRI. CETSA and ITDRF_CETSA_ were utilized to assess the binding between GXNI or DSS and CXCR1 in thermodynamic levels. **(B, C)** WB blots of CETSA and ITDRF_CETSA_. **(D, E)** The curve of CETSA and ITDRF_CETSA_. **(F)** Danshensu-CXCRI interaction is depicted by active binding site (3D & 2D model).

In order to determine the active ingredients in GXNI that interact with CXCR1, the previously identified GXNI ingredients were screened by molecular docking (50). The top 10 GXNI compounds with the highest affinity to interact with CXR1 (-CDOCKER ENERGY score) were Benzoic acid, Danshensu, 4-Hydroxycinnamic acid, Protocatechuic aldehyde, Ferulic acid, Vanillin, Salicylic acid, Furoic acid, Butylphthalide and Rosmarinic acid (**Figure 5A**). Since a previous HPLC-DAD-ESI-MS^n^ qualitative and quantitative analysis suggested that the content of Benzoic acid is extremely low while the Danshensu is known to be the main component in GXNI (44), we consider that Danshensu as a key candidate of GXNI active component inhibiting CXCR1-NF-κB-COX-2/ICAM-1/VCAM-1-mediated IL-8 signaling pathway.

### Verification of GXNI and DSS binding to CXCR1 by CETSA

Thermal Shift Assay (TSA) was performed to evaluate whether GXNI ingredients or Danshensu could directly bind to CXCR1. HL-1 cell lysate, with or without drug (GXNI or Danshensu) treatment, was heated to denature and precipitate the mCXCR1 protein, and then the remaining soluble mCXCR1 was detected *via* using WB. CETSA curve comparison showed that the thermal stability of CXCR1 treated with GXNI or Danshensu was substantially improved compared with the control, and this thermal stabilization between Danshensu and mCXCR1 was dose dependent from ITDRF_CETSA_ (**Figures 5B–E**). Because the intrinsic limitation of the animal model and the above thermal stabilization data did not sufficiently exclude the possible influence of mCXCR2, we further evaluated the affinity of DSS with mCXCR2 and hCXCR1 using CETSA. As shown in **Supplementary Figure 1**, the affinity between DSS and mCXCR1 is stronger than that of mCXCR2, and DSS also binds to hCXCR1 to increase its thermal stability (**Supplementary Figures 1D, E** **)**. Shown in the 3D and 2D interaction diagrams in **Figure 5F**, molecular docking results indicated that the key residues of CXCR1 binding to DSS were SER123, ASN120, HIS207, TRP255 and GLY124. These results not only revealed that the ingredients in GXNI could stably bind to CXCR1, but also confirmed the molecular docking results of Danshensu as the main component of GXNI mediating the GXNI inhibition of the CXCR1-NF-κB-COX-2/ICAM-1/VCAM-1 pathway in MIRI.

### Effect of DSS on HL-1 cells after OGD/R

**Figure 6:**
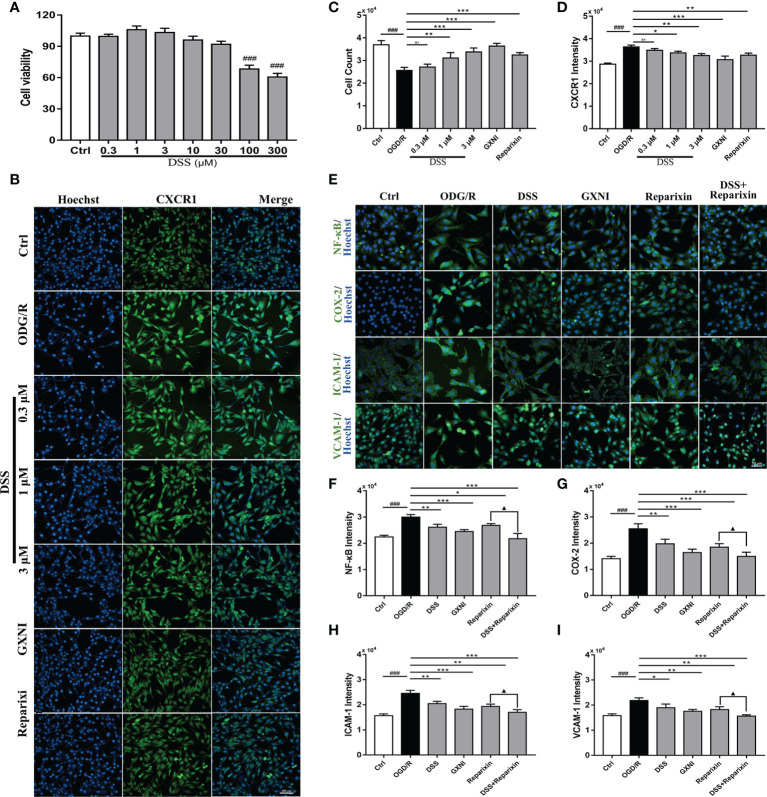
DSS inhibited the expression of NF-κB, COX-2, ICAM-1 and VCAM-1 in HL-1 cells after OGD/R. **(A)** Bar graph quantitation of DSS on the viability of HL-1 cells. After OGD/R, the protective effect of DSS on HL-1 cells and its effect on the expression of CXCR1 protein were evaluated by immunocytofluorescence. **(B)** Representative images of each group, in which the nuclei were stained in blue by Hoechst and the CXCR1 protein was stained green. Scale bar = 100 µm. **(C)** Bar graph quantitation of the number of cells in each group. **(D)** Quantification of the average fluorescence intensity of CXCR1 in each group. **(E)** Representative images of each group. The nucleus was stained blue, and NF-κB, COX-2, ICAM-1 and VCAM-1 were labeled green. Scale bar = 50 µm. **(F)** Quantification of the average fluorescence intensity of NF-κB in each group. **(G)** Quantification of the average fluorescence intensity of COX-2 in each group. **(H)** Quantification of the average fluorescence intensity of ICAM-1 in each group. **(I)** Quantification of the average fluorescence intensity of VCAM-1 in each group. Values were expressed as mean ± SD (n = 3). ^###^ *P* < 0.001 vs. Ctrl group; ^*^ *P* < 0.05, ^**^ *P* < 0.01, ^***^ *P* < 0.001 vs. OGD/R group; ^▲^ *P* < 0.05 vs. Reparixin group. ns: no significance.

To evaluate the effect of DSS on HL-1 cells after OGD/R damage, different concentrations of DSS (0.3 µM - 300 µM) were first tested using the CCK-8 kit. The results indicated that when the DSS concentration reached 10 µM, the cell viability of HL-1 was significantly inhibited (**Figure 6A**). Therefore, 0.3 µM, 1 µM, and 3 µM DSS were used for the follow-up experiments. As shown in **Figures 6B, C**, OGD/R significantly reduced HL-1 cell number (Hoechst nuclear stain), compared to that in the control. Similar to that of Reparixin (100nM), an inhibitor of CXCR1, DSS at concentrations of 1 µM and 3 µM and GXNI at a dose of 1 µL/mL effectively protected HL-1 cells from OGD/R injury while 0.3 µM DSS had no significant effect. At the same time, DSS (1 µM and 3 µM), GXNI and Reparixin markedly reversed the overexpression of CXCR1 protein in HL-1 cells caused by OGD/R injury (**Figures 6B, D** **)**. These results were not only consistent with the results of the *in vivo* experiments, but also suggested that DSS may be the main active ingredient of GXNI to protect HL-1 cardiomyocytes from OGD/R injury by inhibiting the CXCR1 overexpression.

To further elucidate the inhibitory mechanism of DSS on CXCR1 activation, recombinant IL-8 protein (100 nM) was used as an agonist to induce CXCR1/2 activation *in vitro*. One μM DSS and 1 μL/mL GXNI significantly reduced the fluorescence intensity of mCXCR1 and mCXCR2 in HL-1 cells after IL-8 intervention, and the effect was similar to that of Reparixin (**Supplementary Figures 2A–C**). Notably, DSS have a stronger inhibitory effect on mCXCR1 than on mCXCR2 (**Supplementary Figures 2A–C** **)**. Due to the differences of receptor subfamily expression between mouse and human, we also evaluated the effect of DSS on hCXCR1/2 activation in human AC-16 cardiomyocytes, and the results also indicated that 1 μM DSS and 1 μL/mL GXNI evidently reduced the fluorescence intensity of hCXCR1 and hCXCR2, whereas the inhibitory effect of DSS on hCXCR1 is stronger (**Supplementary Figures 2A, D, E** **)**. These findings are also consistent with the RNA-seq results, in which GXNI down-regulated the mRNA level of CXCR1 by more than 30 folds, while only down-regulated CXCR2 by over 9 folds in the myocardial tissue of MIRI mice (**Figure 3H**).

### Effect of DSS on CXCR1-NF-κB-COX-2/ICAM-1/VCAM-1 pathway after OGD/R

To further illustrate the effect of DSS inhibition of CXCR1, downstream proteins of the signaling pathway in HL-1 cells were detected *via* immunocytofluorescence. Compared with the control, the expression levels of NF-κB, COX-2, ICAM-1 and VCAM-1 after OGD/R were significantly increased (**Figures 6E–I**). Similar to CXCR1 inhibitor Reparixin, DSS at 1 µM and GXNI at a dose of 1 µL/mL substantially inhibited the expression of these downstream proteins (**Figures 6E–I**). Interestingly, when DSS and Reparixin were applied in combination, the high expression levels of NF-κB, COX-2, ICAM-1 and VCAM-1 induced by OGD/R were further inhibited compared with that of DSS alone (**Figures 6E–I**). Therefore, these result confirmed that DDS may be a main active component of GXNI down-regulating the CXCR1-NF-κB-COX-2/ICAM-1/VCAM-1 pathway.

### Effects of GXNI and DSS on inflammatory factor release and leukocyte infiltration induced by *in vivo* I/R or *in vitro* OGD/R

**Figure 7:**
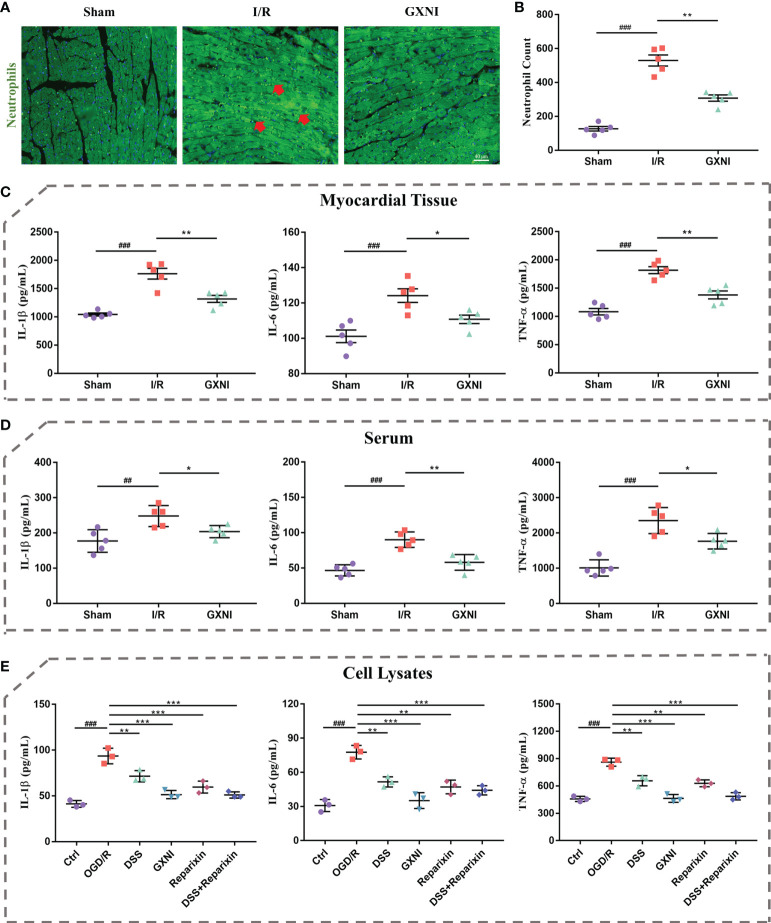
GXNI and DSS inhibited excessive release of inflammatory factors and leukocyte infiltration induced by I/R or OGD/R. **(A)** Representative images of immunohistofluorescence staining of mouse myocardium tissue in the sham, I/R, and GXNI (9 mL/kg) groups. The nuclei were stained in blue, while neutrophils were in green. **(B)** Quantification of neutrophil numbers in the sham, I/R and GXNI (9 mL/kg) groups. **(C)** The expression levels of IL-1β, IL-6 and TNF-α in the supernatant of myocardial tissue of mice in the sham, I/R and GXNI (9 mL/kg) groups. **(D)** The expression levels of IL-1β, IL-6 and TNF-α in the serum of mice in the sham, I/R and GXNI (9 mL/kg) groups. Values were expressed as mean ± SEM (n = 5). ^##^ *P* < 0.01, ^###^ *P* < 0.001 vs. Sham group, ^*^ *P* < 0.05, ^**^ *P* < 0.01 vs. I/R group. Scale bar = 40 µm. **(E)** Quantitation of the expression levels of IL-1β, IL-6 and TNF-α in cell lysates of each group in *in vitro* experiments. Values were expressed as mean ± SD (n = 3). ^###^ *P* < 0.001 vs. Ctrl group; ^**^ *P* < 0.01, ^***^ *P* < 0.001 vs. OGD/R group.

Since GXNI treatment of acute myocardial infarction is mainly related to the anti-inflammatory effects mediated by CXCR1-NF-κB-COX-2/ICAM-1/VCAM-1-mediated IL-8 signaling pathway (**Figures 3E, H**), the release of inflammatory factors and leukocyte infiltration were further examined. Immunohistofluorescence staining results of the *in vivo* mouse tissues showed that the number of myeloperoxidase polyclonal antibody-labeled neutrophils in the myocardial tissue of the I/R group was significantly increased compared with the sham, while GXNI (9 mL/kg) intervention clearly reduced the excessive increase of neutrophils (**Figures 7A, B** **)**. GXNI (9 mL/kg) remarkably reversed the increased expression levels of inflammatory factors such as IL-1β, IL-6 and TNF-α in myocardial tissue and serum of MIRI mice (**Figures 7C, D** **)**. In paralell, the contents of IL-1β, IL-6 and TNF-α *in vitro* HL-1 cells after OGD/R were substantially increased compared with control (**Figure 7E**). Similar to CXCR1 inhibitor Reparixin, GXNI and DSS significantly inhibited the contents of IL-1β, IL-6 and TNF- α in HL-1 cells after OGD/R (**Figure 7E**). Moreover, the combined administration of DSS and Reparixin reduced the contents of IL-1β, IL-6 and TNF-α further in cell lysate. Taken together, these results demonstrated that GXNI and its active ingredient DSS significantly inhibited the excessive inflammatory response induced by I/R or OGD/R, such as the release of inflammatory factors and leukocyte infiltration.

## Discussion

The new findings in this study are as following (1): We demonstrated GXNI as an effective treatment of AMI in a preclinical setting and revealed that more than 3000 genes are regulated by GXNI in MIRI. In particular, the critical GXNI-regulated pathways are found to be IL-8 signaling, Leukocyte extravasation signaling and ILK signaling pathways. (2) The IL-8 signaling pathway, particularly its subordinate branch CXCR1-mediated NF-κB/COX-2/ICAM-1/VCAM-1 signaling pathway, is the main targets of GXNI regulation in MIRI. (3) We identified that Danshensu is a key active ingredient among the GXNI components *via* a direct interaction with CXCR1.

Multi-component and multi-targeting TCM have attracted more and more attention in the treatment of complex diseases (10, 11, 51). Our previous study have shown that GXNI exerts a neuroprotective effect on mice with ischemic stroke *via* regulating SHH-PTCH1-GLI1-mediated axonal guidance signaling in a cerebral I/R model (47). Remarkably, although GXNI also improves myocardial I/R damage of mice with acute myocardial infarction, the detailed mechanisms involved are different. Similarly, another herbal medicine Danhong injection promotes angiogenesis *via* activating the MiR-126/ERK/VEGF pathway to reduce myocardial damage caused by I/R (52), while exerts a neuroprotection effect on ischemic stroke rats mainly by regulating the PI3K-Akt pathway (53). QiShenYiQi dropping pill, yet another herbal medicine containing Danshen, relieves MIRI and CIRI by improving mitochondrial dysfunction and regulating neuroinflammatory network mobilization, respectively (54, 55). In addition, Shuxuening injection not only plays an important role in the treatment of cardiovascular and cerebrovascular diseases, but also has different mechanisms in different pathological stages of the same disease. For example, Shuxuening injection improves I/R injury mainly through anti-inflammatory and antioxidant effects in the acute phase of stroke mice, promotes the recovery of neurological function by inhibiting G-CSF-mediated granulocyte adhesion and diapedesis pathway in the subacute phase, and restores post-stroke cognitive and motor deficits *via* regulating BDNF-mediated Neurotrophin/Trk signaling pathway in the recovery phase (56–58). These results indicated that Chinese herbal medicine with extensive pharmacological effects could specifically regulate certain pathways or targets in the treatment of different diseases caused by I/R injury to achieve a more excellent therapeutic effect.

Recent research advances have shown that CXCR1 is not only a key therapeutic target in many cancers, but also plays an important role in other diseases (59, 60). It has been reported that the activation of CXCR1 could be a “double-edged sword” for cardiovascular diseases. On one hand, interleukin-8 (IL-8) secreted after myocardial ischemia recruits bone marrow-derived mesenchymal stromal cells (MSCs) to the sites of degenerated tissue of myocardium by interacting with CXCR1 overexpressed on MSCs, thereby reducing the ischemic area and improving cardiac function (61). Simultaneously, CXCL8a-CXCR1 signaling is also necessary for the proliferation of coronary artery endothelial cells during cardiac regeneration (62). On the other hand, as a chemokine receptor, CXCR1 “pulls” cells including but not limited to the above cells. For example, CXCR1 also mediates the migration of neutrophils and participates in the cascade events of inflammatory excessive injury (63). These also make it difficult to evaluate the advantages and disadvantages of CXCR1 in the whole AMI process. It is worth noting that previous reports mainly focused on evaluating the changes of CXCR1 level in peripheral blood after AMI and its impact on prognosis. Up till now, there is still a lack of evaluation on the biological functions of CXCR1 and its downstream pathways in cardiomyocytes after AMI, which may be the key to revealing the advantages and disadvantages of CXCR1 in the whole process of AMI. This study not only revealed that CXCR1 is a key target for the treatment of AMI and is regulated by GXNI, but also found that the high expression of CXCR1 in myocardial tissue caused by MIRI exacerbated the inflammatory response *via* activating the downstream COX-2/ICAM-1/VCAM-1 through the NF-κB pathway. As an inflammatory mediator, COX-2 is induced by NF-κB under certain conditions, which promotes the release of inflammatory cytokine (64, 65). It is known that ICAM-1 and VCAM-1 not only play important roles in angiogenesis, but also closely related to leukocyte infiltration (66, 67). It should be noted that these are all pointing out the roles of CXCR1 in cardiomyocytes, rather than directly mediating leukocyte infiltration as a chemokine receptor. Therefore, the overactivation of CXCR1 resulted in more fault than credit in the early stage of AMI according to previous reports and current evidence. Past investigations have revealed significant similarities and differences of cytokine receptor biology between mouse and human (27, 28, 33, 34). The mCXCR1 was characterized as a homologue of hCXCR1. To enhance the clinical implications of our study, we further determined the effect of GXNI and its component DSS on hCXCR1, assessed the affinity of DSS with hCXCR1 using CETSA and evaluated the inhibitory effect of GXNI and DSS on IL-8 activated hCXCR1/2 expression. The binding of DSS to hCXCR1 and the significant inhibition of activated hCXCR1 by DSS offset the inherent limitations of animal models to a certain extent and also suggested that CXCR1 may be an effective target for the treatment of AMI in the clinic.

Although we have revealed and proved that GXNI can play an anti-acute myocardial ischemia-reperfusion injury *via* inhibiting the CXCR1-NF-κB-COX-2/ICAM-1/VCAM-1-mediated IL-8 signaling pathway, there are still some issues that need to be addressed by further investigation. For example (1), in addition to the NF-κB/COX-2 pathway mediated by CXCR1 in the IL-8 signaling pathway, other mechanisms implicated in our RNA-seq data, such as Leukocyte extravasation signaling pathway and ILK signaling pathway for GXNI-regulated genes in I/R heart, remain to be explored by in-depth analysis to clarify their role during GXNI treatment of AMI. (2) Although this study and previous studies (47) have established the therapeutic effects and mechanisms of GXNI in improving MIRI and CIRI, it is still unknown if the same or different active ingredients of GXNI participated in the treatment of these two I/R injuries. (3) Whether there is a common mechanism during GXNI treatment of I/R-mediated brain injury or myocardial injury is still unclear. Future work to explore the GXNI role in cardiac and cerebral I/R injury protection not only may help to understand the multicomponent and multi-target nature and holistic action of TCM, but also contribute to novel drug development targeting the inflammation-immunoregulation network in ischemic heart and brain diseases.

## Conclusion

**Figure 8:**
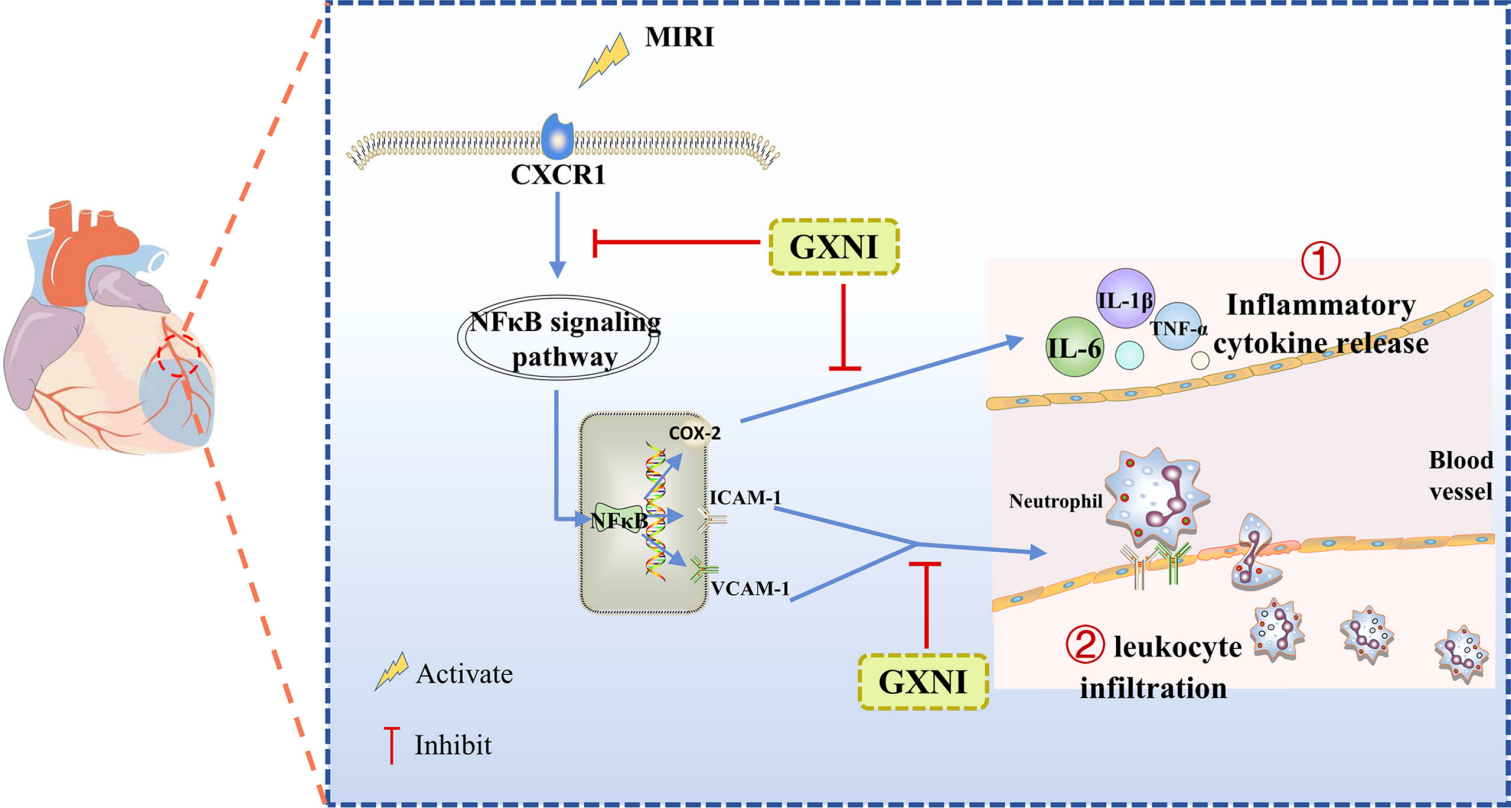
** **A schematic diagram of the mechanism of GXNI ameliorated myocardial injury in I/R mice *via* regulating the CXCR1-NF-κB-COX-2/ICAM-1/VCAM-1 pathway to inhibit the release of inflammatory factors and leukocyte infiltration.

Our study demonstrated that GXNI reduced cardiac dysfunction and myocardial damage caused by I/R injury *via* inhibiting excessive inflammatory factor release and leukocyte infiltration through CXCR1-NF-κB-COX-2/ICAM-1/VCAM-1-mediated IL-8 signaling pathway (**Figure 8**), with Danshensu serving as the key active ingredient by direct interaction with CXCR1.

## Data availability statement

The original contributions presented in the study are publicly available. This data can be found here: https://www.ncbi.nlm.nih.gov/geo/query/acc.cgi?acc=GSE214122.

## Ethics statement

The animal study was reviewed and approved by Laboratory Animal Ethics Committee of Tianjin University of Traditional Chinese Medicine (License number: TCM-LAEC2020107).

## Author contributions

YZ conceptualized and designed the study. GX, ML, JXL, HW, JWL and SH performed the relevant experiments in **Figures 1**-**3**. GX and ML provided **Figures 4**, **5**. GX, JXL and SH completed the RT-PCR and WB experiments and the drawing of **Figure 6**. GX and JXL participated in the relevant experiments in **Figures 7**, **8**. GF and ML helped with the design of the study and the interpretation of the results. YZ and GX wrote the manuscript. All authors contributed to the article and approved the submitted version.

## Funding

This study was supported by grants from National Key R&D Program Project (2018YFC1704502), National Science Foundation of China (NSFC 81873037 and 82104431), and in part by a fund from Shenwei Pharmaceutical Co. Inc.

## Acknowledgments

We would like to thank our lab members, particularly Drs. Yuxin Feng, Jian Yang and Pengzhi Dong, for stimulating discussion and sharing of reagents.

## Conflict of interest

The laboratory has previously received research fund from Shenwei Pharmaceutical Co. Inc. The funder was not involved in the study design, collection, analysis, interpretation of data, the writing of this article or the decision to submit it for publication.

The authors declare that the research was conducted in the absence of any commercial or financial relationships that could be construed as a potential conflict of interest.

## Publisher’s note

All claims expressed in this article are solely those of the authors and do not necessarily represent those of their affiliated organizations, or those of the publisher, the editors and the reviewers. Any product that may be evaluated in this article, or claim that may be made by its manufacturer, is not guaranteed or endorsed by the publisher.
