# Supplementary material for: CXCR1 and its downstream NF-κB inflammation signaling pathway as a key target of Guanxinning injection for myocardial ischemia/reperfusion injury
Source: Front Immunol. 2022 Oct 17;13:1007341. doi: 10.3389/fimmu.2022.1007341 (PMC9618804; doi:10.3389/fimmu.2022.1007341)
Supplement: Supplementary file 1 [file Image_1.pdf]

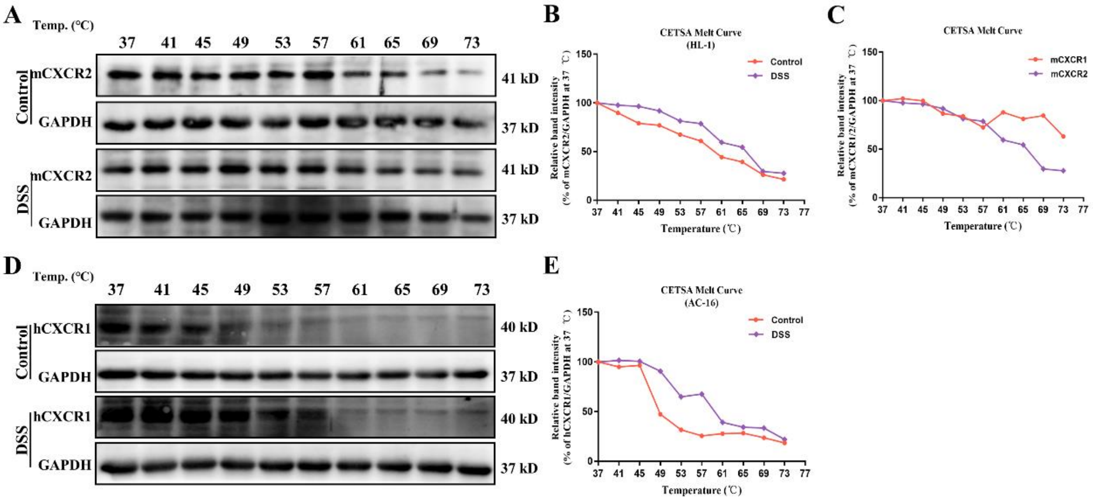

Supplementary Figure 1. Verification of DSS binding to mCXCR2 or hCXCR1 by CETSA. (A, D) WB blots of CETSA. (B-C, E) The curve of CETSA.
