# Supplementary material for: CXCR1 and its downstream NF-κB inflammation signaling pathway as a key target of Guanxinning injection for myocardial ischemia/reperfusion injury
Source: Front Immunol. 2022 Oct 17;13:1007341. doi: 10.3389/fimmu.2022.1007341 (PMC9618804; doi:10.3389/fimmu.2022.1007341)
Supplement: Supplementary file 2 [file Image_2.pdf]

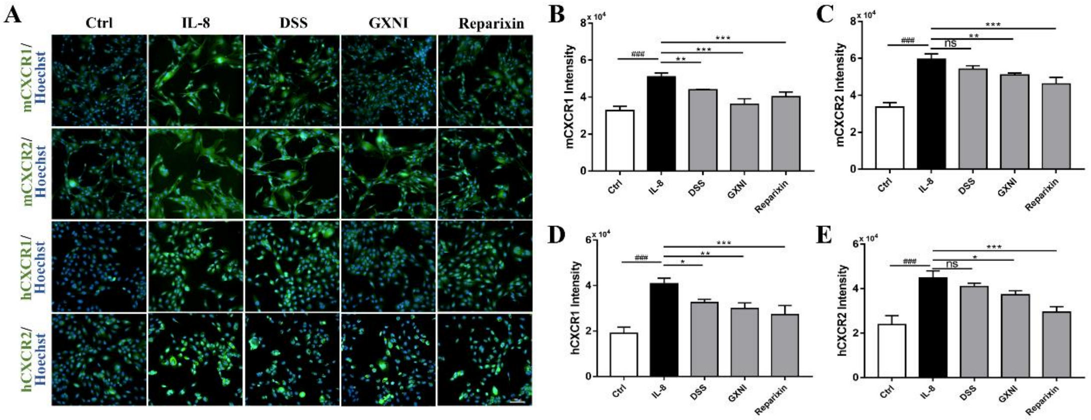

Supplementary Figure 2. Effects of GXNI and DSS on activation of mCXCR1/2 and hCXCR1/2 by IL-8. (A) Representative images of each group, in which the nuclei were stained in blue by Hoechst and the mCXCR1/2 and hCXCR2 were stained green. (B, C) Quantification of the average fluorescence intensity of mCXCR1/2 in each group. (D, E) Quantification of the average fluorescence intensity of hCXCR1/2 in each group. Values were expressed as mean ± SD (n = 3). $^{###}$ $P < 0.001$ vs. Ctrl group; $^{*}P < 0.05$, $^{**}P < 0.01$, $^{***}P < 0.001$ vs. IL-8 group. Scale bar = 100 μm.
